# A Comparison of Classification Techniques to Predict Brain-Computer Interfaces Accuracy Using Classifier-Based Latency Estimation†

**DOI:** 10.3390/brainsci10100734

**Published:** 2020-10-14

**Authors:** Md Rakibul Mowla, Jesus D. Gonzalez-Morales, Jacob Rico-Martinez, Daniel A. Ulichnie, David E. Thompson

**Affiliations:** 1 Mike Wiegers Department of Electrical & Computer Engineering, Kansas State University, Manhattan, KS 66506, USA; morales03@ksu.edu (J.D.G.-M.); jacobprico@ksu.edu (J.R.-M.); 2 Department of Biomedical Engineering, Wichita State University, Wichita, KS 67260, USA; danielulich@ksu.edu

**Keywords:** brain-computer interfaces (BCI), classification methods, P300 speller, P3 latency estimation, sparse autoencoders (SAE)

## Abstract

P300-based Brain-Computer Interface (BCI) performance is vulnerable to latency jitter. To investigate the role of latency jitter on BCI system performance, we proposed the classifier-based latency estimation (CBLE) method. In our previous study, CBLE was based on least-squares (LS) and stepwise linear discriminant analysis (SWLDA) classifiers. Here, we aim to extend the CBLE method using sparse autoencoders (SAE) to compare the SAE-based CBLE method with LS- and SWLDA-based CBLE. The newly-developed SAE-based CBLE and previously used methods are also applied to a newly-collected dataset to reduce the possibility of spurious correlations. Our results showed a significant (p<0.001) negative correlation between BCI accuracy and estimated latency jitter. Furthermore, we also examined the effect of the number of electrodes on each classification technique. Our results showed that on the whole, CBLE worked regardless of the classification method and electrode count; by contrast the effect of the number of electrodes on BCI performance was classifier dependent.

## 1. Introduction

Brain-computer interfaces (BCIs) are an alternative communication technology for people with severe neuromuscular disorders such as amyotrophic lateral sclerosis, cerebral palsy, stroke, or spinal cord injury. BCIs are defined as systems that record brain signals, interpret and translate those signals into an output device to perform user-desired actions [1,2]. One type of BCI is the P300 speller, first introduced by Farwell and Donchin [3], which gained significant attention from BCIs researchers due to its short training period and good performance [4]. As the name suggests, the P300 speller uses the P300 event-related potential (ERP), which is elicited by rare and task-relevant stimuli [5]. In the standard P300 speller system, the user observes different characters and commands in a matrix format and the columns and rows are flashed in a random order. The user will count the number of times the target character is flashed. An oddball paradigm is created due to the low probability of a flashed row/column containing the target, which therefore elicits P300 ERPs.

However, the P300 is not a perfectly stereotypical waveform. Its amplitude and latency vary widely for different users [6], and even for the same user in different sessions [7]. These variations are influenced by many factors, such as age, gender [8], fatigue, exercise [9] and attention [10]. One major effect of P300 latency variation is decreased system performance [11,12].

Several studies have proposed methods to estimate characteristics of the P300 potential including latency (e.g., [13,14]. However, only a few studies have examined the effect of this jitter on P300 speller performance; to our knowledge, the first was our earlier study on classifier-based latency estimation (CBLE) [11]. Later, another independent study also confirmed a negative effect of latency jitter on BCI performance [12]. We also used CBLE estimates and wavelet transforms to provide latency jitter information to a second-level classifier [15]. The combination resulted in an enhanced BCI performance. However, the potential of the CBLE method to predict BCI performance needs to be verified for different classification method and using a different dataset.

CBLE uses the classifier’s sensitivity to latency variability to estimate P300 latency. In our previous work, it was claimed that (i) CBLE is classifier independent and (ii) CBLE can be used to predict BCI accuracy. A comparison of least-squares (LS) and stepwise linear discriminant analysis (SWLDA) was used to support the first statement. However, both LS and SWLDA are linear classifiers, and SWLDA has the same solution subspace with LS for binary classification problems [16,17]. Hence classifier independence was indicated, but not verified, particularly for non-linear classifiers.

The work presented here is a part of a doctoral dissertation [18]. In this work, we will extend our previous CBLE investigation using a sparse autoencoder (SAE), and will examine if classifier independence holds for this non-linear classifier. Both of the previous classification methods (LS, SWLDA) as well as the new non-linear method (SAE) will be used with a new P300 dataset to further verify CBLE’s ability to predict BCI accuracy. The motivation behind choosing these three classification methods are:

(i) LS provided the best overall performance on the dataset used in CBLE’s original article [11],
(ii) In a classifier comparison study [19] SWLDA provided the overall best performance, and
(iii) A recent study [20] showed that SAE provided the best overall performance on their dataset for P300 speller. But SAE has not been used to estimate latency jitter to our knowledge.

## 2. Methods

### 2.1. Experimental Setup

**Figure 1:**
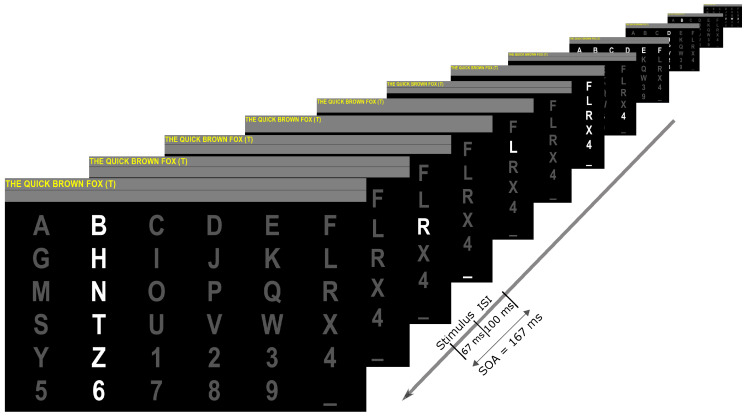
Visual interface of the 6×6 matrix used in this study. A row or column intensified for 67 ms, followed by a 100 ms pause. The front-most image shows an intensification of the column containing the character “T”. This is the current target, so a P300 is expected to be elicited by this intensification.

Data were collected from each participant in three sessions, i.e., on three different days, using BCI2000’s [21] row-column P300 speller paradigm. Each session was comprised of copying three sentences. For each sentence, each row/column was either intensified or replaced with Einstein’s face for 67 ms (stimulus duration) with an inter-stimulus interval of 100 ms. The stimulus onset asynchrony (SOA) was therefore 167 ms. A complete set of 12 intensification or replacements is called a sequence. Figure 1 shows a visualization of the stimulus presentation for a sequence. For each character, we recorded data for 10 sequences. The copied sentences are shown in Table 1. The data from the first sentence in session 01 was used as training data to train the online classifiers and the data for remaining sentences were used as test data. The bolded sentences (one for each session) used Albert Einstein’s iconic tongue face image instead of flashing.

EEG data were recorded using a Cognionics Mobile-72 EEG system with a sampling frequency of 600 Hz. The Mobile-72 EEG system is a high-density mobile EEG system with active Ag/AgCl electrodes placed according to the modified 10-20 system. Reference and ground were on the right and left mastoids, respectively.

### 2.2. Participants

Nine healthy volunteers participated in this study. Data from two participants have been excluded due to their poor online and offline performance. Among the remaining participants, six were male and one female, with an average age of 20.86±4.56 years. Two participants had previous brain-computer interface experience. Participants were provided informed consent and the recording process was performed in accordance with Kansas State University’s Institution Review Board (IRB) protocol No. 8320.

### 2.3. EEG Pre-Processing

Data were filtered using a finite impulse response (FIR) bandpass filter with corner frequencies at (0.5–70.0) Hz, then split into epochs of 750 ms post-stimulus. The epochs were then downsampled by a factor of 30 using a moving average and downsample operation.

Two different sets of electrodes were used for classification. The first set was all 64 electrodes, while the other set was composed of 32 electrodes selected based on data from each participant. To select the electrodes, the average P300 ERPs was produced by taking the difference of the average responses to target and non-target epochs on the training data. The power spectral density (PSD) of the resulting average ERP was used to select the 32 channels with the largest 3 Hz signal power (which should include the P300 response).

### 2.4. Classification Strategy

Detecting the presence of the P300 ERP is a binary classification problem, and most classifiers use the following general equation:

(1) $$\hat{y}\left(x\right)={\hat{w}}^{T}.f\left(x\right)+b$$

where x is the feature vector, w is the weight vector and f(.) is the transformation function. This transformation function f(.) can be a nonlinear function, linear function, or simple identity function. For example, the sparse autoencoder classifier uses a logistic sigmoid function. $\hat{y}\left(x\right)$ is called the classifier’s “score”, and is used to decide the class of each “observation" or measurement. Since we expect the presence of P300 for one row and one column in each sequence, the target character is selected by

(2) $$\begin{array}{c} \hat{R}=\arg\max_{r}\sum_{r=1}^{6}\sum_{s=1}^{S}\hat{y}\left(x_{\mathrm{row}}\right) \end{array}$$

(3) $$\begin{array}{c} \hat{C}=\arg\max_{c}\sum_{c=1}^{6}\sum_{s=1}^{S}\hat{y}\left(x_{\mathrm{col}}\right) \end{array}$$

Here $\hat{R}$ and $\hat{C}$ are the predicted row and column, respectively. *S* is the number of sequences for each character. This classification strategy prevails in the P300 classification literature and is used in numerous studies (e.g., [19,22]).

#### 2.4.1. Classifier-Based Latency Estimation (CBLE)

Standard P300 classification uses a single time window (e.g., 0 ms to 800 ms post-stimulus [19]) time-locked to each stimulus presentation. The Classifier-Based Latency Estimation (CBLE) method [11] uses many time-shifted copies of the post-stimulus epochs, and finds the time shift that corresponds to the maximum score. The statistical variance of the CBLE is denoted vCBLE and is used as the predictor of the BCI’s performance. In this study, BCI accuracy is predicted for each participant using the vCBLE estimates of that participant and the regression coefficients of the relationship between vCBLE and accuracy. The regression coefficients are obtained from the relationship between vCBLE and accuracy from all other participants (i.e., equivalent to leave-one-participant-out cross validation).

#### 2.4.2. Least Squares (LS)

LS is a linear classifier, meaning that it works by taking a weighted sum of the inputs (features).

(4) $$\hat{y}\left(x\right)={\hat{w}}_{LS}^{T}\left[x\;1\right]$$

where ${\hat{W}}_{LS}$ is estimated from the training data and corresponding class labels (y) using the following equation:

(5) $${\hat{W}}_{LS}={\left(X^{T}X\right)}^{-1}X^{T}y$$

#### 2.4.3. Step-Wise Linear Discriminant Analysis (SWLDA)

Step-Wise Linear Discriminant Analysis (SWLDA) is an extension of Fisher’s linear discriminant [23] and was found very effective for P300 classification [24]. SWLDA trains a linear discriminant analysis (LDA) classifier using a stepwise forward and backward regression method. Based on the F-test statistic, the step-wise method progressively adds the most correlated features in the discriminant model and removes the least correlated features during the forward and backward regression, respectively. LDA finds the optimal features using the following equations:

(6) $$\begin{array}{cc} & \hat{y}\left(x\right)=w^{T}\left(x-x_{0}\right) \end{array}$$

where

(7) $$\begin{array}{c} w=\Sigma^{-1}\left(\mu_{1}-\mu_{0}\right), \end{array}$$

(8) $$\begin{array}{c} x_{0}=\frac{1}{2}\left(\mu_{1}+\mu_{0}\right)-\left(\mu_{1}-\mu_{0}\right)\frac{\log(\pi_{1}/\pi_{0})}{{\left(\mu_{1}-\mu_{0}\right)}^{T}\Sigma^{-1}\left(\mu_{1}-\mu_{0}\right)} \end{array}$$

where Σ is the covariance matrix, π is the prior probability of membership in each class, and μ is the mean vector. In our case, we used *p* <0.05 as a threshold to consider a feature statistically significant, and *p* >0.10 to remove the least significant features. Also, the maximum number of features to be included was restricted to 60 features according to [24].

#### 2.4.4. Sparse Autoencoder

A single autoencoder(AE) is a fully-connected, two-layer neural network model which consists of one encoding layer and one decoding layer. The dimension of the encoding layer is the same as the dimension of the input features. The dimension of the decoding layer is, in general, less than the dimension of the encoding layer. The task of an AE is to encode the input features (*x*) to a hidden representation (*z*) with the aim to later reconstruct the input features (*x*) from *z* by minimizing the reconstruction error. For an input vector *x*, the encoder layer maps the vector *x* to another vector *u* such that

(9) $$\bar{u}=f^{\left(1\right)}\left(W^{\left(1\right)}\bar{x}+{\bar{b}}^{\left(1\right)}\right)$$

here, *f* is the transfer function of the encoder, *W* is the weight matrix, *b* is the bias vector and the superscript ${*}^{\left(1\right)}$ denotes layer 1. In our work, we will use a modified version of AE which is commonly known as sparse autoencoders (SAE). In SAE, sparsity is induced by adding a regularizer term to the cost function to limit over-fitting. The sparsity regularization [25] term, $\Omega_{sparsity}$ is defined by the using the Kullback-Leibler divergence of the average activation value, ${\hat{\rho }}_{i}$ of a neuron *i* and its desired value, ρ,

(10) $$\begin{array}{c} \Omega_{sparsity}=\sum_{j=1}^{L}KL\left(\rho ∥{\hat{\rho }}_{i}\right)\\ =\sum_{j=1}^{L}\rho \log\frac{\rho }{{\hat{\rho }}_{i}}+\left(1-\rho \right)\log\frac{1-\rho }{1-{\hat{\rho }}_{i}} \end{array}$$

here, *L* is the number of the neuron in the hidden layer. Kullback-Leibler divergence [26] is a measure of how similar or different two distributions are. Adding the sparsity regularization term requires ρ and ${\hat{\rho }}_{i}$ to be very similar to minimize the cost function. Another regularization, known as $L_{2}$ regularization, is also used to prevent $\Omega_{sparsity}$ from becoming small due only to higher values of weights. $L_{2}$ regularization, $\Omega_{weights}$ is defined as:

(11) $$\begin{array}{c} \Omega_{weights}=\sum_{i=1}^{L}\sum_{j=1}^{N}\sum_{k=1}^{D}w_{jk}^{2} \end{array}$$

here, *N* is the number of observations and *D* is the dimension of the input (number of variables). Then the sparse autoencoder method uses the following cost function to estimate the parameters:

(12) $$J\left(w,b\right)=\frac{1}{N}\sum_{j=1}^{N}\sum_{k=1}^{D}{\left(x_{dn}-{\hat{x}}_{dn}\right)}^{2}+\lambda \Omega_{weights}+\beta \Omega_{sparsity}$$

where λ is the $L_{2}$ regularization coefficient and β is the sparsity regularization coefficient. The SAE decoding layer reconstructs the input features and attempts to minimize the cost function shown in Equation (12). Once the SAE is trained, the decoding layer is removed and the encoded features are used as input to a softmax classifier. Softmax classifiers are a generalized version of the logistic classifier, and provide the probability that input features belong to certain class.

(13) $$\hat{y}\left(x\right)=p\left(y=1|z\right)=\frac{e^{z^{T}w_{1}}}{\sum_{i=1}^{2}e^{z^{T}w_{i}}}$$

These probabilities are treated as the classifier scores as mentioned in the Equation (1).

#### 2.4.5. Parameter Selection

LS has no parameters to optimize, and SWLDA parameters were selected from the literature [24]. This work used 200 hidden units with λ=0.004, β=4. We empirically chose the number of hidden units and the values of regularization coefficients. We also investigated the performance of stacked-SAEs (i.e., multiple layers of sparse autoencoders) and found negligible or no improvement in spelling performance. During the investigation of stacked-SAEs, we used data from all participants. Given the significant increase in computational complexity with stacked-SAEs, and the corresponding negligible or no improvement in performance, we used single-layer SAEs in this investigation.

### 2.5. Performance Evaluation

To evaluate the classifier performance we have computed the system spelling accuracy on each test sentence. Though the information transfer rate (ITR) [27] or BCI utility metric [28] are commonly used metrics for system performance evaluation, these metrics will only differ in the number of sequences are different for different participants or methods. Since we have used a fixed number of sequences (10 sequences) per character for all participants, a comparison using spelling accuracy will reflect the equivalent comparison using ITR or Utility metric. Comparing ITR or Utility metric for a fixed number of sequences for all participants is redundant if spelling accuracy is reported.

The accuracy for each method will be compared using multiple statistical tests. Firstly, accuracy for each method is compared using the Friedman test [29] to find the difference between accuracy for different methods. The Friedman test [29,30] is the non-parametric alternative to repeated-measures Analysis of Variance (ANOVA) that uses a group ranking method. The Friedman test is recommended method for comparisons between classifiers [31] because of its robustness to outliers and the fact that it does not assume normality of the sample means. If the Friedman test detects a significant difference between the obtained accuracy for different methods, a post-hoc analysis is required to find which pairs in the group have significant differences.

For the post-hoc analysis, we used mean rank based multiple comparison methods [32]. Mean ranks post-test is recommended as post-hoc Friedman test in many articles (e.g., [31,33]) and books [34,35]. However, alternative tests are also suggested in the literature [36]. In [36], they discussed several drawbacks of mean ranks-based post-hoc analysis and suggested to use a sign-test or the Wilcoxon signed-rank test [37] to overcome the identified drawbacks. The Wilcoxon signed-rank test is also suggested as an alternative for comparing two classifiers in [31]. Based on the results of the Friedman test, besides mean ranks based comparison, a post hoc analysis using the Wilcoxon signed ranks test [37] also performed as suggested in [31] for multiple accuracy comparison. In our study, we used the Wilcoxon signed-rank test for multiple comparisons post-hoc analyses, adjusting the *p*-value with the conservative Bonferroni correction method.

For the above statistical analysis, we used MATLAB as the primary analysis platform. For the Friedman test, friedman.m function of the Statistical toolbox was used. For the multiple comparison method, multcompare.m function was used. In case of the Wilcoxon signed-rank test based multiple comparison post-hoc analysis, signrank.m function and a custom MATLAB implementation following the procedure described in [36] were used.

## 3. Results

As explained in Section 2.3, we have assessed BCI performance using two different sets of electrodes. LS(64), SWLDA(64), and SAE(64) will denote the classification results using data from all 64 electrodes, while LS(32), SWLDA(32), and SAE(32) will denote the classification results using data from 32 electrodes.

### 3.1. Friedman Test with Post Hoc Analysis

In our case, the null hypothesis of the Friedman test is “no significant difference between the accuracies of each method”. The Friedman test yielded a *p*-value of <$10^{-17}$, which allowed us to reject the null hypothesis.

**Figure 2:**
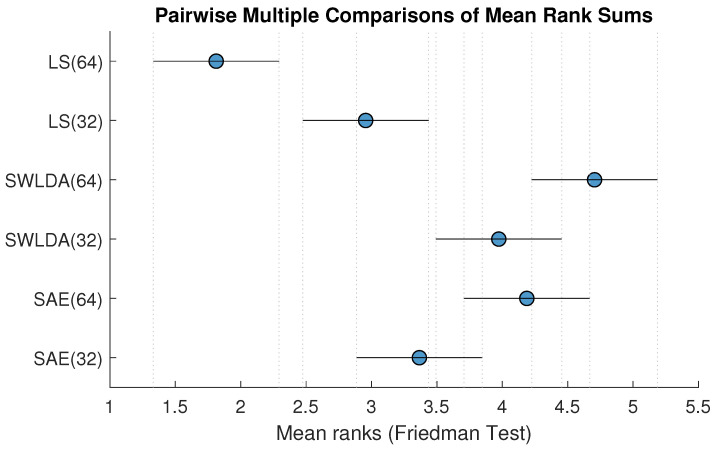
Post hoc analysis: Mean ranks of BCI accuracy with confidence intervals for each methods using multiple comparison method [32]. Higher numerical rank indicates better performance.

Figure 2 shows a graphical representation of the results from the post-hoc analysis. It shows the mean ranks for each method from the Friedman test and the confidence intervals of the ranks from the post-hoc analysis. This figure illustrates the significant or non-significant differences between each method. For instance, the rank of the method LS(64) is significantly lower than the ranks of all other methods. The mean rank of SWLDA(64) is significantly better than the rank of LS(64), LS(32), and SAE(32).

### 3.2. Wilcoxon Signed-Ranks Test

Table 2 shows the *p*-values of pairwise multiple comparisons using the Wilcoxon signed-ranks test. The effect of the number of electrodes and the classification methods are reported in Section 3.3 and Section 3.4, respectively, based on the results showed in Figure 2 and Table 2.

### 3.3. Effect of Number of Electrodes

All three classification methods were examined using EEG recordings from all electrodes and a reduced number of electrodes. Here, we will report the statistical test results for all channels vs. the reduced number of channels. From the Table 2,

1. LS: The accuracy using all channels is significantly worse than using a reduced set of channels.
2. SWLDA: The set of all channels performed better than the reduced channel set, but the difference was not significant.
3. SAE: The set of all channels performed better than the reduced channel set, with the difference close to but above the usual significance threshold (adjusted *p* = 0.068, below 0.05 without Bonferroni correction).

### 3.4. Effect of Classification Method

Here we will focus on the differences between different classification methods from Figure 2 and Table 2. We compared the best-performing channel set for each method to ensure a fair comparison. Therefore, results for LS(32) were compared to SWLDA(64) and SAE(64).

1. LS vs. SWLDA: SWLDA significantly outperformed LS (adjusted *p*-value $8.29\times 10^{-5}$). The results from Table 2 and Figure 2 are congruent in this case.
2. SWLDA vs. SAE: SWLDA slightly outperformed SAE, but the different was highly non-significant (*p*-value 1).
3. SAE vs. LS: SAE significantly outperformed LS (adjusted *p*-value 0.0047). The significant difference is also observed in Figure 2.

### 3.5. Relation between BCI Accuracy and P300 Latency Variations

**Figure 3:**
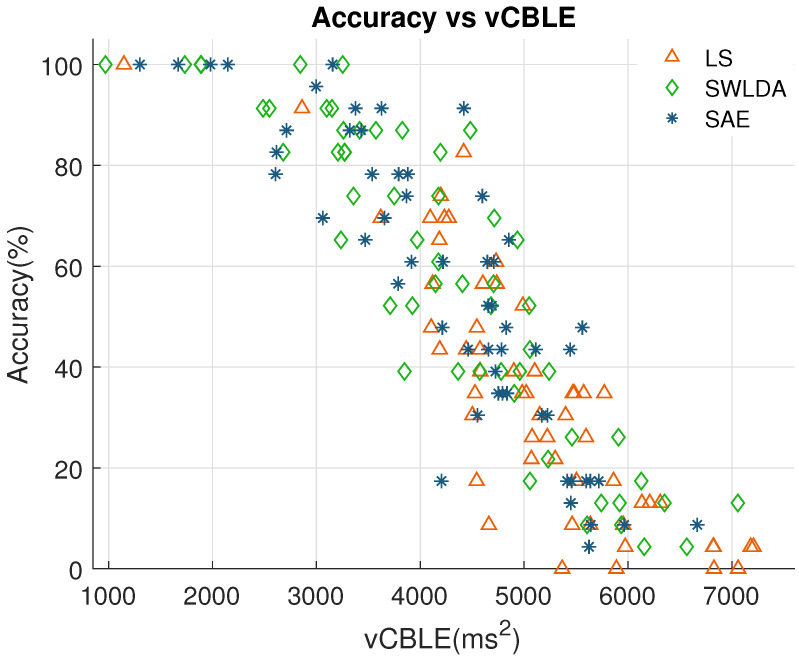
Accuracy plotted against the variance of classifier-based latency jitter estimates (vCBLE) using LS, SWLDA and SAE classifiers.

Figure 3 shows the relationship between BCI accuracy and the variance of CBLE using LS, SWLDA and SAE classifiers. To prevent over-cluttering, Figure 3 includes only results using all electrodes. From this figure, it is evident that BCI performance is highly negatively correlated with the variance of CBLE. The negative correlation is consistent for all three classification methods. For LS, the correlation coefficient is −0.85 ($p<10^{-15}$), for SWLDA correlation coefficient is −0.90 ($p<10^{-20}$), and for SAE correlation coefficient is −0.87 ($p<10^{-17}$).

### 3.6. Predicting BCI Accuracy from vCBLE

**Figure 4:**
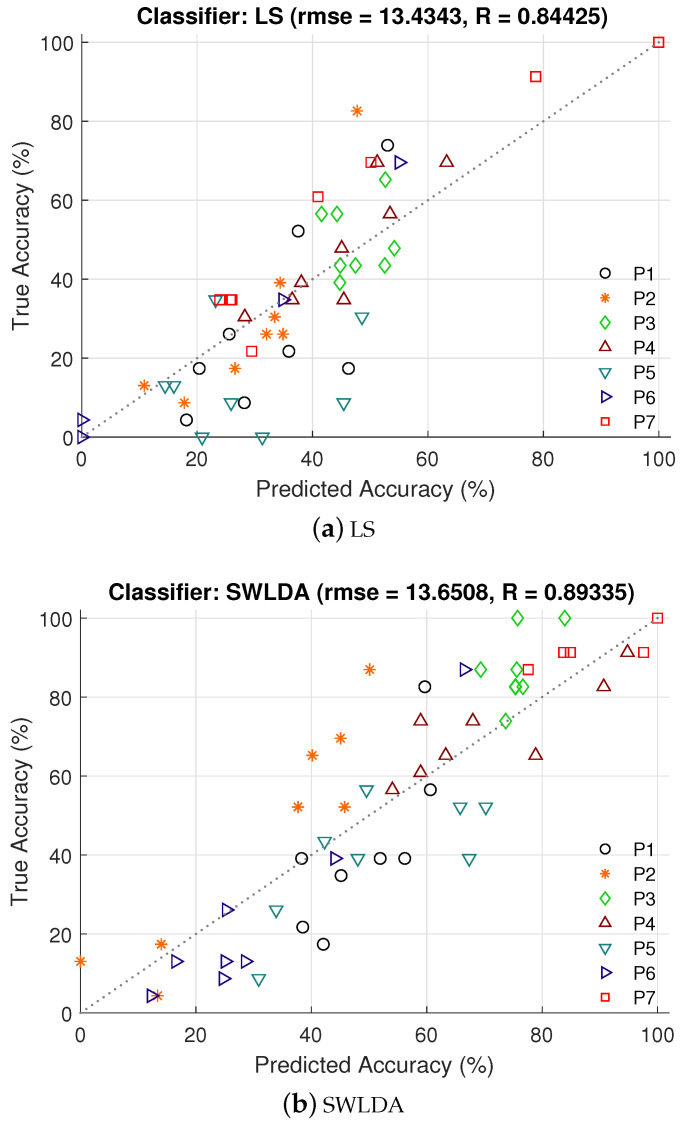

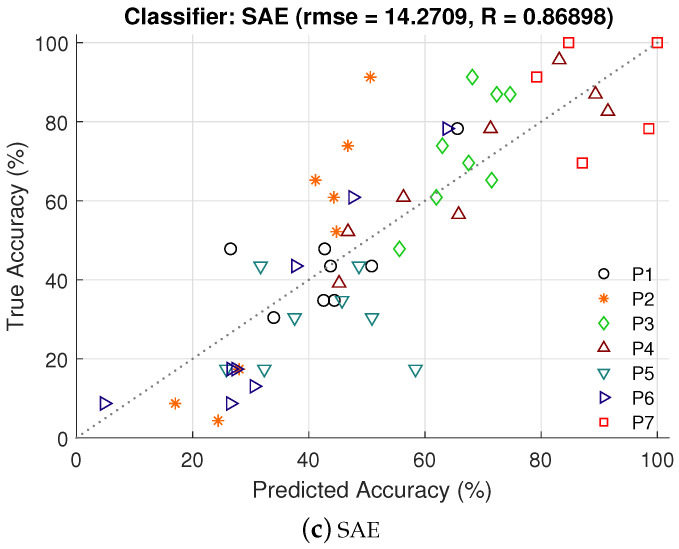
Predicted BCI accuracy from vCBLE are plotted against true accuracy for three different classifiers. P1, P2, P3, P4, P5, P6, and P7 are indicating each participant.

Figure 4 shows the predicted accuracy using variances of CBLE (vCBLE) for LS, LDA, and SAE classifiers, respectively. Predicted accuracy using vCBLE for all the classifiers are significantly correlated with the actual accuracy. The root mean square errors (rmse) for three classifiers are $rmse_{LS}=13.43$, $rmse_{LDA}=13.65$, and $rmse_{SAE}=14.27$, the coefficients of determination are $R_{LS}^{2}=0.713$, $R_{LDA}^{2}=0.798$, and $R_{SAE}^{2}=0.755$. While these metrics leave some room for improvement, the randomness inherent in observing accuracy from a small number of characters prevents reaching perfect prediction. Even for “ideal” prediction (where the system correctly guesses the exact binomial parameter for each dataset), the resulting error would be expected to be $rmse_{ideal}$ = 8.0–8.4 and $R^{2}$ = 0.9–0.93 based on our simulations.

## 4. Discussion

From the results shown in Section 3.3, we observed that the effect of the number of electrodes is classifier-dependent. LS performed better with features from fewer electrodes whereas both SWLDA and SAE performed better with features from all available electrodes (though the SWLDA and SAE effects were not statistically significant). This is consistent with theory—both SWLDA and SAE use inherent feature reduction techniques and should be less prone to the curse of dimensionality.

On our current dataset, the performance of SWLDA is significantly better than the performance of LS classification, which is congruent with the reported findings in [19]. But SAE failed to prove better than the performance of SWLDA. Furthermore, the required training time for SAEs is often outweighing their performance [20]. Overall, SWLDA may be a better choice for P300 speller BCIs in terms of combined performance and practicability.

For our P300 speller dataset, we have observed a high negative correlation between P300 latency jitter and classification accuracy. This finding is consistent with our previously reported results in the earlier CBLE study, as well as the findings reported in another independent study [12].

### 4.1. Limitations

CBLE is based on an assumption that the ERP complex shifts with a single latency which is estimated on a single-trial basis. This prevents any study of latency variation between different ERP components such as P3a and P3b. The same assumption prevents the study of single-trial spatial latency variations, if such variations exist.

### 4.2. Future Work

Predicting BCI accuracy from vCBLE may be further improved by using non-linear modeling to find the relationship between accuracy and vCBLE. In conjunction with vCBLE, other predictive variables (e.g., age, gender, sleeping hours) may also be included for better prediction.

## 5. Conclusions

In this work, we extended the CBLE method for sparse autoencoders (SAE) and used on a newly collected dataset to test the ability to use a measure of the variance of P300 latency to predict classification accuracy in the P300 speller. Our analysis showed that the CBLE method worked similarly with the SAE method.

From the results presented here, we can conclude that the effect of the number of electrodes on performance is relative to the classification methods. LS classification works well with less features (data from fewer electrodes); SWLDA and SAE work well with a higher number of features (data from all available electrodes). Overall, SWLDA was the best classifier on our dataset, and also had the strongest correlation between BCI performance and vCBLE.

The similitude of the results from this dataset and the results reported in the CBLE original work strongly establishes that (i) the P300 BCI system performance is negatively correlated with latency variations, (ii) CBLE can be used to predict BCI accuracy. Moreover, the similar vCBLE and accuracy correlation supports the claim that CBLE is classifier independent.

While collecting this dataset, we used face stimuli in one of the three sentences in each session. Face stimuli are known to have better performance than basic character intensification for P300 speller [38,39]. Our overreaching goal was to determine and compare the variance of P300 latency for character intensification versus face stimuli. However, due to an insufficient number of participants, we could not able to reach that goal. Our future direction on this research will be to collect more data so that we can better understand if face stimuli have any effect on the variance of P300 latency. In the future, we will also aim to determine and compare the variance of P300 latency for other recently developed paradigms such as tactile stimulation [40] based P300.

## Figures and Tables

**Table 1:** Sentences copied by the participants.

Session	Sentence to Spell
	THE QUICK BROWN FOX
01	THANK YOU FOR YOUR HELP
	**THE DOG BURIED THE BONE**
	MY BIKE HAS A FLAT TIRE
02	**I WILL MEET YOU AT NOON**
	DO NOT WALK TOO QUICKLY
	**YES. YOU ARE VERY SMART**
03	HE IS STILL ON OUR TEAM
	IT IS QUITE WINDY TODAY

**Table 2:** Adjusted (Bonferroni correction [32]) *p*-values of pairwise multiple comparisons using Wilcoxon signed-ranks test.

Methods	LS(64)	LS(32)	SWLDA(64)	SWLDA(32)	SAE(64)
LS(32)	1.55 $\times \;10^{-4}$ ***	-	-	-	-
SWLDA(64)	$1.33\times 10^{-8}$ ***	$8.29\times 10^{-5}$ ***	-	-	-
SWLDA(32)	$3.67\times 10^{-6}$ ***	$3.34\times 10^{-4}$ ***	0.543	-	-
SAE(64)	$2.09\times 10^{-8}$ ***	0.0047 **	1	1	-
SAE(32)	$1.77\times 10^{-4}$ ***	1	0.0013 **	0.149	0.068

** Adjusted p<0.01; *** Adjusted p<0.001.

## Abbreviations

The following abbreviations are used in this manuscript:

AE	Autoencoder
ANOVA	Analysis of Variance
BCI	Brain-computer interface
CBLE	Classifier-based latency estimation
ERP	Event-related potential
ITR	Information transfer rate
LDA	Linear discriminant analysis
LS	least-squares
SAE	Sparse autoencoders
SOA	Stimulus onset asynchrony
SWLDA	Stepwise linear discriminant analysis
vCBLE	Statistical variance of the CBLE
